# What is the future of axillary surgery for breast cancer?

**DOI:** 10.3332/ecancer.2013.319

**Published:** 2013-05-16

**Authors:** M Ahmed, M Douek

**Affiliations:** Department of Research Oncology, King’s College London, Guy’s Hospital Campus, Great Maze Pond, London, SE1 9RT, UK

**Keywords:** sentinel lymph node biopsy, axillary lymph node dissection, magnetic resonance imaging, ultrasmall paramagnetic iron oxide

## Abstract

The Z11 trial demonstrated a subgroup of patients with low axillary burden who do not benefit from axillary lymph node dissection (ALND) at short-term follow-up when treated with adjuvant whole-breast radiotherapy and systemic therapy. We consider the role of sentinel lymph node biopsy (SLNB) and look at and beyond the Z11 trial to consider further imaging studies, which may offer truly minimally invasive management of the axilla and relegate SLNB to the realms of history.

Regional lymph node status provides information regarding staging, local control, and prognostic outcomes in all cancers. This information was provided in breast cancer by axillary lymph node dissection (ALND). This changed with the development of sentinel lymph node biopsy (SLNB) [1, 2]. Sentinel lymph nodes (SLNs) are defined as the first lymph nodes receiving lymphatic drainage from the primary tumour and therefore the most likely to harbour metastatic cancer via lymphatic spread. SLNB is now the standard of care in patients with a clinically and radiologically clear axilla in early-stage breast cancer.

## The role of SLNB in patients with a clinically and radiologically negative axilla:

SLNB has replaced ALND in patients with a clinically and radiologically negative axilla. The SLN is the most likely node to be positive in the presence of axillary metastases, and if it is positive, there is a 14-fold greater likelihood of non-sentinel lymph node (non-SLN) involvement [3]. A systematic review of 69 trials involving 8059 patients undergoing SLNB validated by ALND demonstrated a 96% success rate for identifying the SLN and a mean false-negative rate of 7.3% [4]. This has been confirmed by five randomised controlled trials comparing SLNB followed by ALND with SLNB followed by ALND only if metastases were present in the SLNB specimens [5–9]. The axillary recurrence rates are less than 0.5% in SLN-negative patients [3, 10]. Therefore, in patients with a clinically and radiologically negative axilla, SLNB is the standard for staging of the axilla. However, in 70% of patients, the SLNB is negative and therefore surgery could be avoided if better axillary mapping techniques were available.

## Patients with a positive SLNB

### Patients with positive SLNs who did not undergo completion ALND

Evidence has accumulated from studies of the outcomes of patients with positive SLNB who did not proceed to completion ALND. Studies have demonstrated that the axillary recurrence rate is low [11–14]. Naik *et al *[11] reported 1.4% axillary recurrence rate at a median follow-up of 31 months in 210 patients with SLN-positive cancer who declined ALND. Three further studies demonstrated no cases of axillary recurrence at a range between 27.6 and 32 months of follow-up [12–14]. These studies demonstrate that there are patients with SLN-positive cancer who have a low axillary burden and do not experience axillary recurrence with the omission of completion ALND and therefore in whom completion ALND is unnecessary.

### The significance of micrometastases

Retrospective studies using serial sectioning and immunohistochemistry (IHC) suggest that micrometastases are prognostically significant [15–18]. However, the ACOSOG ZOO10 [19] and NSABP-32 [20] trials failed to demonstrate any reduction in overall survival in patients found to be SLN positive on IHC but not on H&E at five- and eight-year follow-up, respectively. The retrospective study of 377 patients with a single micrometastatic SLN who did not undergo completion ALND by Galimberti *et al *[21] found that the five-year overall survival was 97.3% and the incidence of axillary recurrence only 1.6%. The report from the extensive SEER database [22] suggested that the amount of completion ALND for positive SLNB, particularly micrometastases, has been falling with no apparent effect upon local control or survival.

Therefore, evidence has accumulated that patients with micrometastatic disease do not need to proceed to completion ALND. This led to posing the partially answered question of which SLN-positive patients actually require a completion ALND. This resulted in the concept of the development of a prospective trial in order to address this emerging trend. 

## ACOSOG Z0011 Trial

The aim of the ACOSOG Z11 trial [23] was to determine whether ALND is beneficial for survival or locoregional control in breast-conserving surgery with whole-breast radiotherapy and systemic therapy in patients with SLN metastases. It was a prospective phase III study that commenced in 1999, enrolling 891 women with clinically T1- or T2-invasive breast cancer without clinical lymphadenopathy and one or two SLNs containing metastases, which were identified by touch preparation, frozen section, or H&E staining. Patients were ineligible if their SLN metastasis was only identifiable using IHC, or if they had three or more positive SLNs. All patients were treated with breast-conserving surgery and tangential whole-breast radiotherapy. No third-field irradiation was given to the axilla. Patients with SLN metastases on SLNB were randomised either to ALND or to no further axillary treatment.

The trial closed early in 2004 due to slow accrual and a lower than anticipated event rate. Patients were followed up for a median of 6.3 years. In the group of patients randomised to ALND, the five-year overall survival and disease-free survival was 91.8% and 82.2%, respectively, whereas in the SLNB-alone group, it was 92.5% and 83.9%. The hazard ratio for treatment-related overall survival was 0.87 after adjusting for age and adjuvant therapy, and it was concluded that no significant difference was observed between the two groups.

### Issues with the Z11 trial

The planned sample size was not achieved due to a lower than anticipated event rate, but the statistical analysis of Z11 was able to demonstrate the non-inferiority of SLNB alone for overall survival with a *p-value *of 0.008. The 95% confidence intervals of the hazards ratio for survival did not cross 1.3, the predefined point at which the treatments would be considered unequal, suggesting the results would not change with a larger sample size [24].

The study has a relatively short-term follow-up. In NSABP B04, the median time to axillary recurrence was 12 months in clinically node-negative women not undergoing ALND [25]. However, the NSABP B04 group did not receive any radiotherapy to the axilla compared with the Z11 study in which all patients underwent whole-breast radiotherapy, where standard opposing tangential fields will irradiate much of level I and II axillary lymph nodes, meaning that the two groups are not truly comparable. In studies by Martelli *et al *[26] and Greco *et al* [27] with populations of largely ER-positive patients comparable with those in Z11, the median times to axillary recurrence were 33.0 and 30.6 months, respectively. Therefore, the Z11 authors would argue that the median follow-up period of 6.3 years is sufficient to capture the majority of axillary recurrences. However, if we look at the results of the Guy’s breast conservation trial [28, 29], consisting of 629 patients with early breast cancer, randomised to either radical mastectomy and postoperative radiotherapy or wide local excision and postoperative radiotherapy, we may want to reconsider this viewpoint. At ten years of follow-up, while there was a significant difference between both local and distant recurrence for the radical mastectomy and wide local excision groups (7% versus 25%, respectively), there was no difference in overall survival (80% in both groups). It was only at 25 years of follow-up that the divergence developed and there was a statistically significant increase in breast cancer deaths in the breast conservation group compared with the mastectomy group (48% versus 38%, *p=0.0016*). This does raise the point that the median 6.3-year follow-up in the Z11 trial may be inadequate to demonstrate differences in overall survival, which will only become prominent with longer-term follow-up.

All patients with heavy axillary burden were excluded from Z11. However, no attempt was made to determine the axillary burden preoperatively using radiological assessment of the axilla, in contrast to the standard practice of the modern management of the axilla. There were a very high proportion of patients who possessed micrometastases, 120 out of 436 in the ALND group, and 160 out of 420 in the SLNB-only group. We know that the large-scale studies have shown that in patients with micrometastases, there are no significant differences in axillary recurrence or overall survival for patients who undergo SLNB only versus completion ALND [19–22]. We also know that for patients with low axillary burden, fewer ALNDs were being performed before Z11, suggesting a more surgically conservative approach to the management of the axilla prior to publication of Z11 [30].

Adjuvant systemic therapy was delivered to 423 out of 436 in the SLNB-only arm, and 403 out of 420 in the ALND arm. Adjuvant systemic therapy is known to diminish locoregional recurrence in breast cancer patients [31]. All patients in Z11 underwent whole-breast radiotherapy. It is known that standard opposing tangential fields will irradiate the SLNB operative field, and by placing the deep field edge 2-cm below the chest wall/lung interface, the entire axillary dissection field site (levels I and II) can be included in nearly all patients [32]. It is therefore likely that significant portions of the axilla were treated in patients in both arms of the Z11 trial.

### Conclusions to be drawn from the Z11 trial

Z11 demonstrates the existence of a group of patients with low axillary burden who do not benefit from completion ALND when combined with whole-breast radiotherapy and systemic therapy. When put in this context, the conclusion does not appear radically remarkable, especially considering the high numbers of patients with micrometastatic disease in both arms. We can conclude that Z11 does support the already existing evidence on the treatment of patients with low axillary burden in the form of micrometastases. It reaffirms that such patients do not benefit from completion ALND as was already supported by current evidence [19–22]. Therefore, one can safely conclude that this group when treated in combination with whole-breast radiotherapy and systemic therapy can avoid proceeding to completion ALND. However, we have to consider whether the numbers of patients with macrometastatic disease (283 in the ALND and 218 in the SLNB-alone arm) are large enough to gain any insight into their safe long-term management. We would suggest that the findings are promising, however, full confidence of the implementation of the Z11 findings will only come with the longer-term follow-up of a larger cohort of patients with macrometastatic disease to ensure there is no late divergence in recurrence and overall survival in these patients. Where does this leave us for managing patients with macrometastatic SLN disease right now? Our opinion is that those patients fitting the Z11 criteria with macrometastatic SLN disease would benefit from being made fully aware of the findings of the study and the fact that conclusions have been drawn from a small cohort over a relatively short follow-up period who all received whole-breast radiotherapy and systemic therapy. If they are happy to undergo these adjuvant therapies and take on the uncertainty, we would be happy to proceed with omitting completion ALND from their management.

## BEYOND Z11

### New trials:

Trials are now necessary to focus upon patients with a clinically and radiologically clear axilla who were excluded from the Z11 trial. This includes those not suitable for conservational breast surgery. This would allow us to determine whether the findings of Z11 could be reproduced in this group of patients. Currently, Z11 data would have to be applied with great caution to patients undergoing mastectomy since they may not receive adjuvant radiotherapy as was the case in all Z11 patients. Patients undergoing neoadjuvant chemotherapy could also be assessed. All patients undergoing neoadjuvant chemotherapy would undergo SLNB before commencing chemotherapy. Those that demonstrated low axillary burden would then be eligible for the study. Subgroups would have to be established for those patients who would undergo mastectomy and those who responded adequately to allow conservative breast surgery. These patients would be randomised in to either observation alone or an ALND group. The third group to be assessed could include patients undergoing breast conservative surgery without whole-breast radiotherapy. This study could involve women over the age of 70 suitable for breast conservative surgery. All patients would undergo breast conservative surgery and SLNB and those with a low axillary burden would be eligible for the study. These patients would then be randomised between observation alone and ALND. Such trials would build on the information accrued from the Z11 dataset rather than replicating it.

### A bloodless future for staging the axilla?

It has been demonstrated that there are patients with a low axillary burden who do not gain additional benefit from ALND. Therefore, if ALND is becoming increasingly infrequent, what is the future role of SLNB? If we are pursuing a less invasive approach, is it not time to consider non-invasive modalities to assess the axilla? Magnetic resonance imaging (MRI) provides an alternative to SLNB in determining axillary burden. Johnson *et al *[33] administered ultrasmall paramagnetic iron oxide (USPIO) periareolarly and performed standard SLNB. The 13 nodes containing metastases had variable quantities of iron within them, but the iron was not present in the area of the node containing the metastasis. Therefore, non-homogenous enhancement of the SLNs and non-SLNs on contrast-enhanced MRI may indicate a metastatic focus. This technique would allow identification of patients with heavy axillary involvement preoperatively and direct progression to ALND. Meng *et al *[34] assessed the cost-effectiveness of MRI and PET for the evaluation of axillary lymph nodes in early breast cancer. They found that the most cost-effective strategy was the replacing of SLNB with MRI. Under this strategy, true positive patients will be correctly diagnosed by MRI and undergo ALND rather than two sequential surgical procedures (SLNB followed by ALND), and true negative patients will be correctly diagnosed without the need for SLNB. The difficulty of applying this on clinical grounds arose in the study due to the false-positive rate for MRI being 6.3% versus 0.2% for the current baseline strategy of SLNB. Harnan *et al *[35] in their systematic review considering the use of MRI assessment of axillary lymph node status in early breast cancer recorded a mean sensitivity and specificity of 90% and 95%, respectively. The highest mean sensitivity and specificity being recorded for USPIO enhanced MRI, with values of 98% and 96%, respectively.

A study that is using ultrasound for the preoperative staging of the axilla is the sentinel node versus observation after axillary ultrasound trial (SOUND) [36]. This is a prospective randomised controlled multicentre study, where patients with breast cancers less than 2-cm in size, suitable for conservative breast surgery, and SLNB will undergo preoperative axillary ultrasound to rule out suspicious nodal involvement. Patients showing a single doubtful (not suspicious) lymph node will undergo an ultrasound-guided fine needle aspiration cytology. Patients either with negative cytology of the single doubtful lymph node or with negative ultrasound of the axilla will be eligible for randomisation into two groups: (arm 1) SLNB ± ALND or (arm 2) no axillary surgical staging. In arm 1, no axillary dissection will be performed either in the case of negative SLNB or in the presence of isolated tumour cells or micrometastases. SLNB will be completed by axillary dissection in the presence of macrometastases diagnosed on SLNB. The primary endpoint is distant disease-free survival with secondary endpoints of distant and axillary recurrences, disease-free survival, and overall survival. This study may address the significance of avoidance of axillary surgery in these patients and the ability of preoperative ultrasound to identify patients with clinically relevant nodal burden, as metaanalyses [37] have demonstrated median ultrasound sensitivity and specificity of only 61.4% and 82.0%, respectively.

## Conclusion

The axillary burden reflects the biology of the tumour. Ideally, the extent of axillary surgery should be guided by the likely axillary tumour burden. The concept of selective axillary surgery has also been suggested, and clearly selective surgery would rely upon the development of improved preoperative imaging modalities. This would allow truly individualised treatments, which take into consideration the patient’s axillary burden in order to optimise their management. The future for USPIO contrast-enhanced MRI in this field is promising. It is essential that we prioritise funding to develop further imaging studies to evaluate preoperative axillary MRI to identify standardised criteria for MRI parameters that define metastatic axillary lymph nodes. The current SentiMAG multicentre trial [38], which is a prospective phase II nonrandomised clinical trial to compare SLNB using magnetic nanoparticles with the standard technique, co-ordinated from Guy’s Hospital has an MRI sub-protocol. This will evaluate preoperative axillary MRI (with USPIO magnetic tracer) for sentinel node imaging and characterisation and ex-vivo MRI with a 9.4-T high-resolution scanner to identify metastases. The aim of the subprotocol is to evaluate MRI for both preoperative and intraoperative detections of lymph node metastases.

The results from such studies will allow us to move forward to a truly minimally invasive approach to the future axillary management of breast cancer and eventually, perhaps, even relegate the SLNB to the pages of history.

## Conflict of interest statement

The authors have no disclosures to make concerning financial and personal relationships with other people or organizations that could inappropriately influence their work.
